# (*E*)-3-(2-Fur­yl)-1-(2-hydroxy­phen­yl)prop-2-en-1-one

**DOI:** 10.1107/S1600536808031644

**Published:** 2008-10-22

**Authors:** Lingqian Kong, Yanhong Liu

**Affiliations:** a Dongchang College, Liaocheng University, Liaocheng 250059, People’s Republic of China; b Liaocheng No.3 Middle School, Liaocheng, People’s Republic of China

## Abstract

In the title mol­ecule, C_13_H_10_O_3_, an intra­molecular O—H⋯O hydrogen bond influences the mol­ecular conformation, and the benzene and furan rings form a dihedral angle of 8.35 (7)°. Weak inter­molecular C—H⋯O hydrogen bonds link mol­ecules into sheets parallel to the *bc* plane.

## Related literature

For a related crystal structure, see: Li *et al.* (1992 ▶).
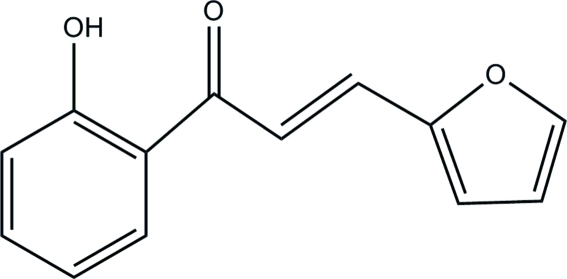


## Experimental

### 

#### Crystal data

C_13_H_10_O_3_
*M* *_r_* = 214.21
Monoclinic, 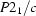

*a* = 3.8560 (5) Å
*b* = 15.6565 (14) Å
*c* = 17.309 (2) Å
β = 95.065 (2)°
*V* = 1040.9 (2) Å^3^
*Z* = 4
Mo *K*α radiation
μ = 0.10 mm^−1^
*T* = 298 (2) K
0.27 × 0.25 × 0.07 mm

#### Data collection

Siemens SMART CCD area-detector diffractometer
Absorption correction: multi-scan (*SADABS*; Sheldrick, 1996 ▶) *T*_min_ = 0.974, *T*_max_ = 0.993
5153 measured reflections
1848 independent reflections
668 reflections with *I* > 2σ(*I*)
*R*_int_ = 0.126

#### Refinement

*R*[*F* ^2^ > 2σ(*F* ^2^)] = 0.067
*wR*(*F* ^2^) = 0.184
*S* = 0.81
1848 reflections
146 parameters
H-atom parameters constrained
Δρ_max_ = 0.22 e Å^−3^
Δρ_min_ = −0.19 e Å^−3^

### 

Data collection: *SMART* (Siemens, 1996 ▶); cell refinement: *SAINT* (Siemens, 1996 ▶); data reduction: *SAINT*; program(s) used to solve structure: *SHELXS97* (Sheldrick, 2008 ▶); program(s) used to refine structure: *SHELXL97* (Sheldrick, 2008 ▶); molecular graphics: *SHELXTL* (Sheldrick, 2008 ▶); software used to prepare material for publication: *SHELXTL*.

## Supplementary Material

Crystal structure: contains datablocks I, global. DOI: 10.1107/S1600536808031644/cv2448sup1.cif

Structure factors: contains datablocks I. DOI: 10.1107/S1600536808031644/cv2448Isup2.hkl

Additional supplementary materials:  crystallographic information; 3D view; checkCIF report

## Figures and Tables

**Table 1:** Hydrogen-bond geometry (Å, °)

*D*—H⋯*A*	*D*—H	H⋯*A*	*D*⋯*A*	*D*—H⋯*A*
O3—H3⋯O2	0.82	1.84	2.544 (4)	144
C1—H1⋯O2^i^	0.93	2.59	3.400 (5)	146
C3—H3*A*⋯O3^ii^	0.93	2.59	3.504 (5)	169

Symmetry codes: (i) 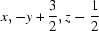; (ii) 
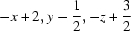
.

**Fig. 1.**
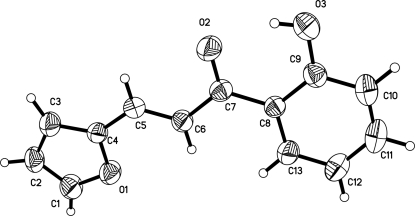
The molecular structure of (I) showing the atomic numbering and 40% probability displacement ellipsoids.

## supplementary crystallographic information

### Comment

In continuation of our ongoing program directed to the development of environmentally benign methods of chemical synthesis, we describe in this paper a user-friendly, solvent-free protocol for the synthesis of chalcones starting from the fragrant aldehydes and fragrant ketones in the presence of NaOH under solvent-free conditions. Using this method, which can be considered as a a general method for the synthesis of chalcones, we obtained the title compound, (I). We present here its crystal structure.

In (I) (Fig. 1), the bond lengths and angles are normal and comparable to those observed in related compound (Li *et al.*, 1992). The benzene and furan rings form a dihedral angle of 8.35 (7)°. In the crystal, weak intermolecular C—H···O hydrogen bonds (Table 1) link the molecules into sheets parallel to *bc* plane.

### Experimental

Furan-2-carbaldehyde (0.3 mmol) and 2-hydroxylacetophenone (0.3 mmol), NaOH (0.3 mmol) were mixed in 50 ml flash under sovlent-free conditions After stirring for 5 min at 373 K, the mixture was soilden slowly and afforded the title compound, then recrystallized from ethanol, affording the title compound as a colourless crystalline solid. Elemental analysis: calculated for C_13_H_10_O_3_: C 72.90, H 4.71%; found: C 72.88, H 4.65%.

### Refinement

All H atoms were placed in geometrically idealized positions (O—H 0.85 Å, C—H 0.93 Å) and treated as riding, with *U*_iso_(H) = 1.2 *U*_eq_(C,O).

### Crystal data

**Table:**

C_13_H_10_O_3_	*F*(000) = 448
*M**_r_* = 214.21	*D*_x_ = 1.367 Mg m^−^^3^
Monoclinic, *P*2_1_/*c*	Mo *K*α radiation, λ = 0.71073 Å
*a* = 3.8560 (5) Å	Cell parameters from 438 reflections
*b* = 15.6565 (14) Å	θ = 2.4–18.4°
*c* = 17.309 (2) Å	µ = 0.10 mm^−^^1^
β = 95.065 (2)°	*T* = 298 K
*V* = 1040.9 (2) Å^3^	Block, colourless
*Z* = 4	0.27 × 0.25 × 0.07 mm

### Data collection

**Table:**

Siemens SMART CCD area-detector diffractometer	1848 independent reflections
Radiation source: fine-focus sealed tube	668 reflections with *I* > 2σ(*I*)
graphite	*R*_int_ = 0.126
Detector resolution: φ and ω pixels mm^-1^	θ_max_ = 25.0°, θ_min_ = 1.8°
φ and ω scans	*h* = −4→4
Absorption correction: multi-scan (SADABS; Sheldrick, 1996)	*k* = −7→18
*T*_min_ = 0.974, *T*_max_ = 0.993	*l* = −20→20
5153 measured reflections	

### Refinement

**Table:**

Refinement on *F*^2^	Primary atom site location: structure-invariant direct methods
Least-squares matrix: full	Secondary atom site location: difference Fourier map
*R*[*F*^2^ > 2σ(*F*^2^)] = 0.067	Hydrogen site location: inferred from neighbouring sites
*wR*(*F*^2^) = 0.184	H-atom parameters constrained
*S* = 0.81	*w* = 1/[σ^2^(*F*_o_^2^) + (0.0721*P*)^2^] where *P* = (*F*_o_^2^ + 2*F*_c_^2^)/3
1848 reflections	(Δ/σ)_max_ < 0.001
146 parameters	Δρ_max_ = 0.22 e Å^−^^3^
0 restraints	Δρ_min_ = −0.19 e Å^−^^3^

### Special details

**Table:**

Geometry. All e.s.d.'s (except the e.s.d. in the dihedral angle between two l.s. planes) are estimated using the full covariance matrix. The cell e.s.d.'s are taken into account individually in the estimation of e.s.d.'s in distances, angles and torsion angles; correlations between e.s.d.'s in cell parameters are only used when they are defined by crystal symmetry. An approximate (isotropic) treatment of cell e.s.d.'s is used for estimating e.s.d.'s involving l.s. planes.
Refinement. Refinement of *F*^2^ against ALL reflections. The weighted *R*-factor *wR* and goodness of fit *S* are based on *F*^2^, conventional *R*-factors *R* are based on *F*, with *F* set to zero for negative *F*^2^. The threshold expression of *F*^2^ > σ(*F*^2^) is used only for calculating *R*-factors(gt) *etc*. and is not relevant to the choice of reflections for refinement. *R*-factors based on *F*^2^ are statistically about twice as large as those based on *F*, and *R*- factors based on ALL data will be even larger.

### Fractional atomic coordinates and isotropic or equivalent isotropic displacement parameters (Å^2^)

**Table:**

	*x*	*y*	*z*	*U*_iso_*/*U*_eq_	
O1	1.0561 (7)	0.73465 (16)	0.48262 (15)	0.0794 (9)	
O2	0.8017 (8)	0.83794 (18)	0.74522 (15)	0.0885 (11)	
O3	0.5477 (10)	0.9672 (2)	0.80912 (17)	0.1030 (12)	
H3	0.6546	0.9220	0.8087	0.154*	
C1	1.1454 (12)	0.6735 (3)	0.4327 (2)	0.0842 (15)	
H1	1.1472	0.6811	0.3794	0.101*	
C2	1.2297 (11)	0.6018 (3)	0.4699 (3)	0.0769 (13)	
H2	1.3018	0.5510	0.4485	0.092*	
C3	1.1887 (11)	0.6177 (3)	0.5478 (2)	0.0748 (13)	
H3A	1.2271	0.5788	0.5882	0.090*	
C4	1.0842 (10)	0.6991 (2)	0.5544 (2)	0.0584 (10)	
C5	0.9983 (9)	0.7493 (2)	0.6177 (2)	0.0608 (11)	
H5	1.0222	0.7240	0.6665	0.073*	
C6	0.8860 (10)	0.8300 (2)	0.6137 (2)	0.0598 (11)	
H6	0.8676	0.8578	0.5661	0.072*	
C7	0.7918 (10)	0.8754 (2)	0.6822 (2)	0.0600 (11)	
C8	0.6700 (9)	0.9648 (2)	0.6757 (2)	0.0553 (10)	
C9	0.5559 (11)	1.0064 (3)	0.7399 (2)	0.0690 (12)	
C10	0.4467 (12)	1.0901 (3)	0.7358 (3)	0.0835 (14)	
H10	0.3697	1.1170	0.7790	0.100*	
C11	0.4529 (13)	1.1329 (3)	0.6679 (3)	0.0930 (16)	
H11	0.3769	1.1893	0.6649	0.112*	
C12	0.5685 (12)	1.0951 (3)	0.6033 (3)	0.0839 (14)	
H12	0.5747	1.1256	0.5573	0.101*	
C13	0.6745 (11)	1.0117 (3)	0.6079 (2)	0.0702 (12)	
H13	0.7518	0.9858	0.5642	0.084*	

### Atomic displacement parameters (Å^2^)

**Table:**

	*U*^11^	*U*^22^	*U*^33^	*U*^12^	*U*^13^	*U*^23^
O1	0.111 (3)	0.0632 (19)	0.0648 (18)	0.0106 (16)	0.0132 (16)	−0.0022 (16)
O2	0.138 (3)	0.067 (2)	0.0625 (18)	0.0095 (18)	0.0190 (18)	0.0029 (15)
O3	0.150 (4)	0.087 (3)	0.076 (2)	0.010 (2)	0.033 (2)	−0.0105 (18)
C1	0.112 (4)	0.076 (3)	0.065 (3)	0.008 (3)	0.015 (3)	−0.014 (3)
C2	0.080 (4)	0.057 (3)	0.095 (3)	0.008 (2)	0.012 (3)	−0.016 (3)
C3	0.086 (4)	0.061 (3)	0.078 (3)	0.008 (2)	0.011 (2)	−0.004 (2)
C4	0.065 (3)	0.053 (2)	0.058 (2)	−0.002 (2)	0.010 (2)	−0.001 (2)
C5	0.063 (3)	0.059 (2)	0.061 (2)	0.001 (2)	0.010 (2)	0.004 (2)
C6	0.067 (3)	0.058 (2)	0.054 (2)	0.002 (2)	0.005 (2)	−0.003 (2)
C7	0.064 (3)	0.057 (3)	0.059 (2)	−0.005 (2)	0.004 (2)	0.001 (2)
C8	0.055 (3)	0.053 (2)	0.058 (2)	−0.0020 (19)	0.006 (2)	−0.001 (2)
C9	0.071 (3)	0.069 (3)	0.068 (3)	−0.001 (2)	0.009 (2)	−0.009 (2)
C10	0.080 (4)	0.073 (3)	0.095 (4)	0.008 (3)	0.001 (3)	−0.023 (3)
C11	0.093 (4)	0.061 (3)	0.122 (4)	0.014 (3)	−0.014 (3)	−0.013 (3)
C12	0.099 (4)	0.063 (3)	0.086 (3)	0.000 (3)	−0.008 (3)	0.007 (3)
C13	0.078 (3)	0.056 (3)	0.076 (3)	−0.004 (2)	0.007 (2)	−0.005 (2)

### Geometric parameters (Å, °)

**Table:**

O1—C1	1.354 (4)	C6—C7	1.455 (5)
O1—C4	1.357 (4)	C6—H6	0.9300
O2—C7	1.237 (4)	C7—C8	1.476 (5)
O3—C9	1.349 (4)	C8—C13	1.386 (5)
O3—H3	0.8200	C8—C9	1.393 (5)
C1—C2	1.321 (5)	C9—C10	1.377 (6)
C1—H1	0.9300	C10—C11	1.354 (5)
C2—C3	1.394 (5)	C10—H10	0.9300
C2—H2	0.9300	C11—C12	1.374 (6)
C3—C4	1.343 (5)	C11—H11	0.9300
C3—H3A	0.9300	C12—C13	1.368 (5)
C4—C5	1.413 (5)	C12—H12	0.9300
C5—C6	1.335 (5)	C13—H13	0.9300
C5—H5	0.9300		
			
C1—O1—C4	106.9 (3)	O2—C7—C8	120.1 (4)
C9—O3—H3	109.5	C6—C7—C8	120.1 (3)
C2—C1—O1	110.8 (3)	C13—C8—C9	117.1 (4)
C2—C1—H1	124.6	C13—C8—C7	122.6 (3)
O1—C1—H1	124.6	C9—C8—C7	120.3 (4)
C1—C2—C3	106.0 (4)	O3—C9—C10	116.6 (4)
C1—C2—H2	127.0	O3—C9—C8	122.0 (4)
C3—C2—H2	127.0	C10—C9—C8	121.4 (4)
C4—C3—C2	108.2 (4)	C11—C10—C9	119.2 (4)
C4—C3—H3A	125.9	C11—C10—H10	120.4
C2—C3—H3A	125.9	C9—C10—H10	120.4
C3—C4—O1	108.2 (3)	C10—C11—C12	121.6 (4)
C3—C4—C5	133.3 (4)	C10—C11—H11	119.2
O1—C4—C5	118.5 (3)	C12—C11—H11	119.2
C6—C5—C4	125.7 (3)	C13—C12—C11	118.8 (4)
C6—C5—H5	117.1	C13—C12—H12	120.6
C4—C5—H5	117.1	C11—C12—H12	120.6
C5—C6—C7	121.6 (3)	C12—C13—C8	122.0 (4)
C5—C6—H6	119.2	C12—C13—H13	119.0
C7—C6—H6	119.2	C8—C13—H13	119.0
O2—C7—C6	119.7 (3)		

### Hydrogen-bond geometry (Å, °)

**Table:**

*D*—H···*A*	*D*—H	H···*A*	*D*···*A*	*D*—H···*A*
O3—H3···O2	0.82	1.84	2.544 (4)	144
C1—H1···O2^i^	0.93	2.59	3.400 (5)	146
C3—H3A···O3^ii^	0.93	2.59	3.504 (5)	169

Symmetry codes: (i) *x*, −*y*+3/2, *z*−1/2; (ii) −*x*+2, *y*−1/2, −*z*+3/2.
